# ALKBH5-mediated m^6^A demethylation of FOXM1 mRNA promotes progression of uveal melanoma

**DOI:** 10.18632/aging.202371

**Published:** 2021-01-10

**Authors:** Lili Hao, Jiayang Yin, Hong Yang, Chaoxuan Li, Linxin Zhu, Lian Liu, Jingxiang Zhong

**Affiliations:** 1 Department of Ophthalmology, The First Affiliated Hospital of Jinan University, Guangzhou 510632, Guangdong Province, China

**Keywords:** uveal melanoma, ALKBH5, FOXM1, m^6^A demethylation

## Abstract

In this study, we found that ALKBH5, a key component of the *N*^6^-methyladenosine (m^6^A) methyltransferase complex, was significantly elevated in uveal melanoma (UM) cell lines and that ALKBH5 downregulation inhibited tumor growth *in vivo*. High ALKBH5 expression predicted worse outcome in patients with UM. EP300-induced H3K27 acetylation activation increased ALKBH5 expression. Downregulation of ALKBH5 inhibited UM cell proliferation, migration, and invasion and increased apoptosis *in vitro*. Besides, ALKBH5 may promote UM metastasis by inducing epithelial-to-mesenchymal transition (EMT) via demethylation of *FOXM1* mRNA, which increases its expression and stability. In sum, our study indicates that AKLBH5-induced m^6^A demethylation of *FOXM1* mRNA promotes UM progression. Therefore, AKLBH5 is a potential prognostic biomarker and therapeutic target in UM.

## INTRODUCTION

Uveal melanoma (UM), which originates from melanocytes, is the most common primary intraocular malignancy in adults [1]. Although the primary tumor can be treated with resection, radiation therapy, and enucleation, up to 50% of UM patients will develop metastatic disease [2, 3]. The most common sites of UM metastasis are the liver (60.5%), lungs (24.4%), skin/soft tissue (10.9%), and bone (8.4%) [4]. Unfortunately, the 1-year survival rate of UM patients with metastases is only 15% [5]. Therefore, there is a pressing need to find useful prognostic biomarkers and therapeutic targets for this disease.

Although previous studies have revealed that distinct genetic alterations occur during the formation and progression of UM [6–8], genomic epigenetic modifications, such as histone modification, DNA modification, and RNA methylation, also play critical roles in the tumorigenesis of UM [8–10]. N6-methyladenosine (m^6^A) methylation is the most prevalent modification in RNA, including mRNAs, LncRNAs, and circRNAs [11]. m^6^A modification is installed by a multicomponent methyltransferase complex consisting of METTL3, METTL14, and WTAP and is erased by FTO and ALKBH5. Moreover, specific RNA-binding proteins, such as YTHDF1, YTHDF2, and YTHDF3, bind to the m^6^A motif to modify RNA function [12, 13]. m^6^A modifications have been reported to affect multiple biological processes, such as RNA stability, splicing, transport, subcellular localization and translation efficiency, and RNA-protein interactions [11]. Recently, RNA methylation was shown to inhibit the progression of UM and ocular melanoma (OM), and decreased m^6^A levels were associated with a poor outcome in OM [14]. Conversely, METTL3-mediated m^6^A modification may promote UM progression by targeting c-Met and increasing its expression [15]. Therefore, understanding of the biological function of m^6^A and potential regulatory mechanisms in human UM is still in its infancy.

Tumor metastasis requires a series of events within the tumor microenvironment, including cell proliferation, loss of cellular adhesion, degradation of the extracellular matrix, and cell migration and invasion [16]. In cancer, epithelial-to-mesenchymal transition (EMT) is a key process leading to tumor metastasis and aggression, stemness, and resistance to therapy [17]. m^6^A modifications have been shown to cause tumor progression via EMT and tumorigenesis. METTL3 induced an m^6^A modification in the coding sequence of Snail, a key transcription factor of EMT, which triggered polysome-induced translation of Snail mRNA in cancer cells [18]. In addition, METTL3-provoked m^6^A methylation of ZMYM1 mRNA facilitated EMT and gastric cancer metastasis [19]. Some studies have demonstrated that EMT promotes the metastasis and aggression of UM [20, 21]. However, the effect of m^6^A modifications on EMT in UM has yet to be explored.

Here, we verified that ALKBH5 overexpression promotes UM progression and found that ALKBH5 expression is increased by EP300-induced H3K27 acetylation activation. In addition, ALKBH5 promotes EMT of UM by upregulating FOXM1 expression via demethylating the m^6^A modification and further increasing the stability of FOXM1 mRNA.

## RESULTS

### High ALKBH5 expression is associated with worse prognosis in patients with UM and promotes UM growth *in vivo*

**Figure 1:**
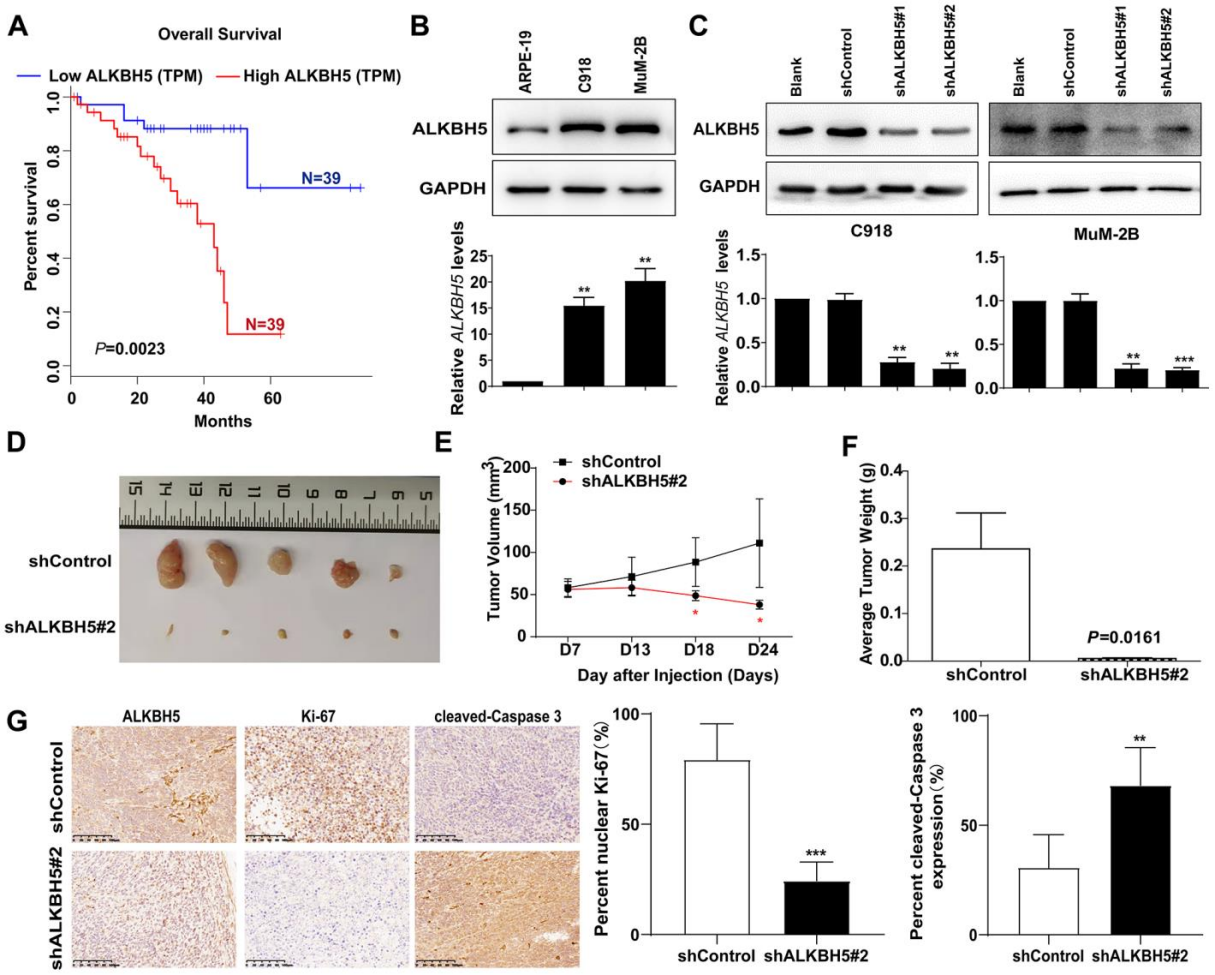
High ALKBH5 expression is associated with worse prognosis in patients with UM and promotes UM growth *in vivo* (**A**) Survival curves of UM patients expressing ALKBH5 at high and low levels in the TCGA cohort from the online GEPIA database (*P* = 0.0023). (**B**) Expression of ALKBH5 protein (upper panel) and mRNA (bottom panel) levels in UM cells was detected by western blot and qRT-PCR. (**C**) ALKBH5 knockdown efficiency was verified at the protein and mRNA levels in C918 (left panel) and MuM-2B (right panel) by western blot and qRT-PCR. (**D**) Knockdown of ALKBH5 effectively inhibited UM subcutaneous tumor growth in nude mice (n=5). (**E**) The growth curves of C918 stably transduced with shALKBH5 in nude mice were significantly dampened compared with those of C918 cells transduced with control plasmid. (**F**) Histogram shows the mean tumor weights from the shALKBH5 and control groups. (**G**) Sections of tumors were stained with anti-ALKBH5, anti-Ki67, and anti-cleaved-Caspase 3 antibodies by IHC staining. Mean ± SEM, t-test, *P < 0.05, **P < 0.01, ***P < 0.001.

To obtain the clinical significance of ALKBH5 expression in patients with UM. The GEPIA database (http://gepia.cancer-pku.cn), which includes the data set from The Cancer Genome Atlas (TCGA), was used to evaluate the prognosis of ALKBH5 expression with a high or low level. As shown in Figure 1A, higher ALKBH5 expression was correlated with worse outcomes in patients with UM.

To investigate the association between ALKBH5 expression and UM, we measured ALKBH5 expression in two human UM cell lines (MuM-2B and C918). As shown in Figure 1B, the protein (Figure 1B, upper panel) and mRNA (Figure 1B, bottom panel) levels of ALKBH5 were significantly elevated in UM cells.

To study the effect of ALKBH5 on UM tumor progression *in vivo*, we used two short hairpin RNAs (shALKBH5#1 and shALKBH5#2) to decrease ALKBH5 expression. As expected, protein (Figure 1C, upper panel) and mRNA (Figure 1C, bottom panel) expression of ALKBH5 was downregulated in MuM-2B and C918 cells that were transfected with two distinct shRNAs. Subsequently, ALKBH5-stable knockdown C918-shALKBH5#2 cells were established and injected subcutaneously into nude mice (Figure 1D, 1E). The results showed that the downregulation of ALKBH5 significantly suppressed tumor growth, as reflected by the tumor volumes (Figure 1E) and weight (Figure 1F) compared with tumors derived from control cells. In addition, IHC staining revealed that, compared with the normal control group, Ki-67 expression was dramatically suppressed and cleaved-caspase3 was increased in the C918-shALKBH5#2 group (Figure 1G). These results demonstrate that ALKBH5 expression is associated with UM tumorigenicity.

### EP300-induced H3K27ac activation promotes ALKBH5 transcription in UM

**Figure 2:**
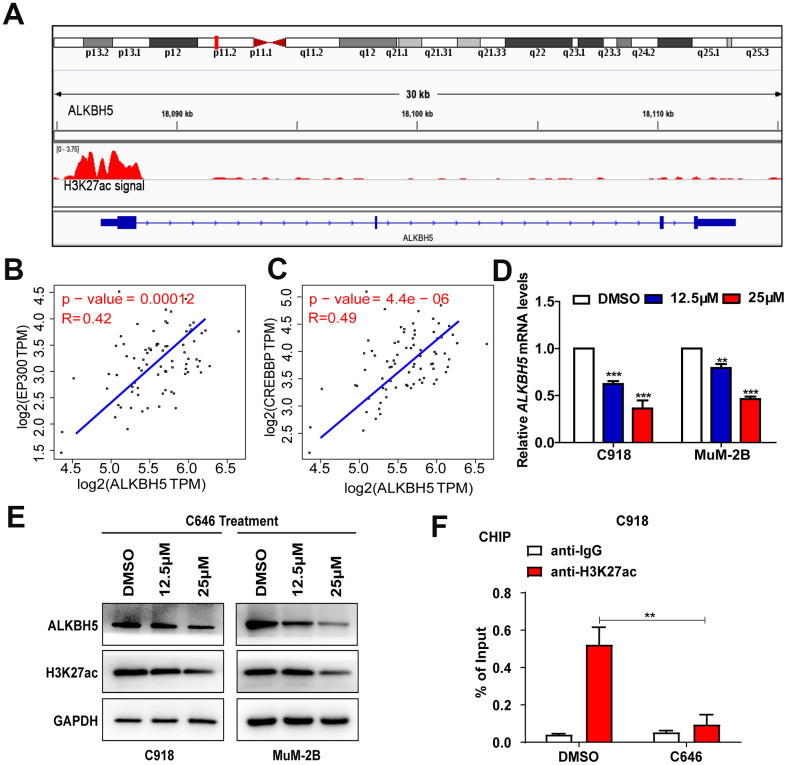
**EP300-induced H3K27ac activation increased ALKBH5 expression in UM.** (**A**) Data from the UCSC Genome Bioinformatics Site (http://genome.ucsc.edu/) showed high enrichment of H3K27ac in the promoter of ALKBH5 in melanoma. (**B**, **C**) ALKBH5 mRNA levels were positively correlated with EP300 (**B**) and CREBBP (**C**) in the UM data set from the TCGA database. (**D**–**E**) The mRNA (**D**) and protein (**E**) levels of ALKBH5 and H3K27ac were decreased when UM cells were treated with C646 for 24 hours. (**F**) ChIP assays were used to determine the enrichment of H3K27ac at the promoter of ALKBH5 after treating C918 cells with C646 and DMSO at 12.5μM for 12 hours. Mean ± SEM, t-test, *P < 0.05, **P < 0.01, ***P < 0.001.

To determine the cause of high ALKBH5 expression in UM cells, we analyzed melanoma data from the UCSC Genome Bioinformatics Site (http://genome.ucsc.edu) and found high enrichment and overlap of H3K27ac peaks at the promoter region of ALKBH5 in melanoma (Figure 2A), which suggests that ALKBH5 may be regulated by chromatin acetylation. It has been reported that H3K27ac is catalyzed by cAMP response element binding protein (CREB) binding protein (CREBBP) and EP300 in a bromodomain-dependent fashion [22]. By analyzing the TCGA data set, we found that EP300 (Figure 2B) and CREBBP (Figure 2C) mRNA expression was positively correlated with ALKBH5 expression in UM.

To verify the relationship between H3K27ac and ALKBH5, we treated UM cells with C646, the histone acetyltransferase inhibitor targeting EP300, and SGC-CBP30, a histone acetyltransferase inhibitor targeting CREBBP. The mRNA (Figure 2D) and protein (Figure 2E) levels of ALKBH5 were significantly decreased in C918 and MuM-2B cells after treating the cells with C646 for 24 hours. Although SGC-CBP30 decreased the expression of H3K27ac, ALKBH5 was not affected (Supplementary Figure 1). Furthermore, the ChIP assay results indicated that the promoter region of ALKBH5 was enriched in EP300 binding and H3K27ac signals, and inhibition of EP300 by C646 treatment could significantly decrease the enrichment of H3K27ac signals in the promoter of ALKBH5 (Figure 2F). These results indicate that EP300-induced H3K27ac activation increases ALKBH5 expression in UM.

### ALKBH5 inhibition decreases proliferation and facilitates cell cycle arrest in UM cells

**Figure 3:**
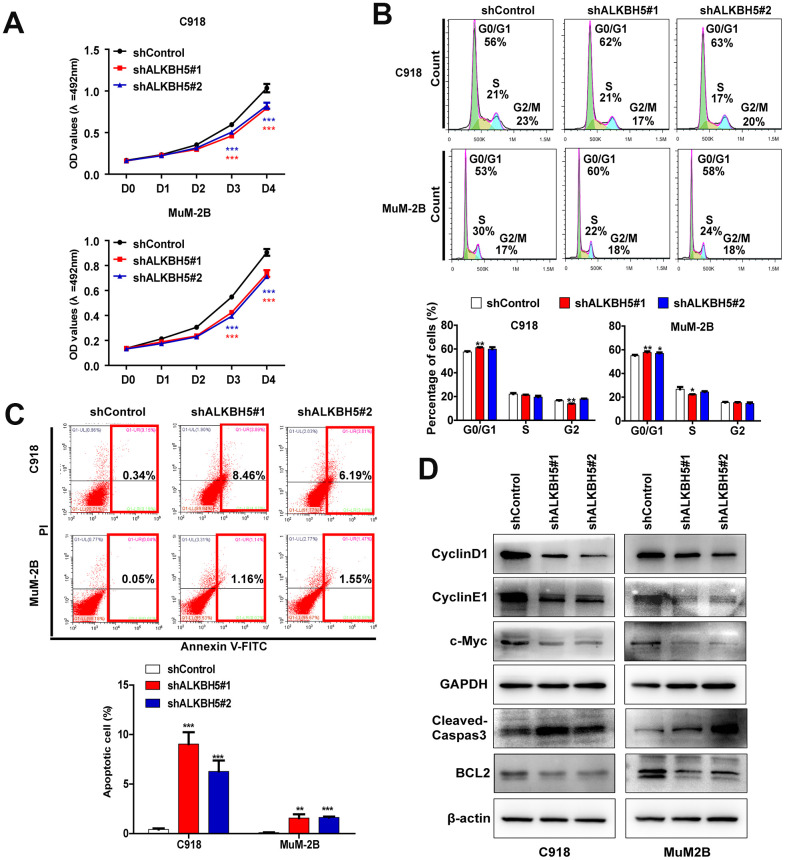
**ALKBH5 knockdown inhibited UM cell growth.** (**A**) Knockdown of ALKBH5 suppressed cell proliferation in UM cells as determined by MTT assay. (**B**) ALKBH5 shRNA treatment induced cell cycle arrest in G1/S phase in UM cells. (**C**) ALKBH5 elicited an apoptotic response in UM cells as determined by annexin V-PITC and PI staining and flow cytometry. (**D**) Western blot showed increased expression of apoptotic related protein, cleaved caspase-3, and decreased expression of BCL-2, c-Myc, cyclin D1 and cyclin E1 in ALKBH5-knockdown UM cells. Mean ± SEM, t-test, *P < 0.05, **P < 0.01, ***P < 0.001.

We performed a series of *in vitro* biological experiments to further assess the function of ALKBH5 in UM cells. Loss of ALKBH5 caused a significant decrease in cell viability in MuM-2B and C918 cells (Figure 3A) and induced G1 to S phase transition arrest (Figure 3B). The protein expression of the G1 to S phase checkpoint regulators cyclin D1 and cyclin E1 was downregulated in shALKBH5-transfected cells compared with the control vector-transfected cells (Figure 3D). These results confirm that ALKBH5 facilitates cell growth by promoting cell cycle progression in UM cells.

### Loss of ALKBH5 promotes UM cell apoptosis

To further explore the effect of ALKBH5 on UM growth, we assessed the effect of ALKBH5 on cell apoptosis using flow cytometry after staining with annexin V and PI. Downregulation of ALKBH5 significantly increased cell apoptosis in MuM-2B and C918 cells (Figure 3C). Moreover, loss of ALKBH5 in MuM-2B and C918 cells significantly increased apoptosis marker protein levels, cleaved caspase-3, and decreased the expression of BCL-2, which is an antiapoptosis marker (Figure 3D).

### Silencing ALKBH5 reduces migration and invasion of UM cells

**Figure 4:**
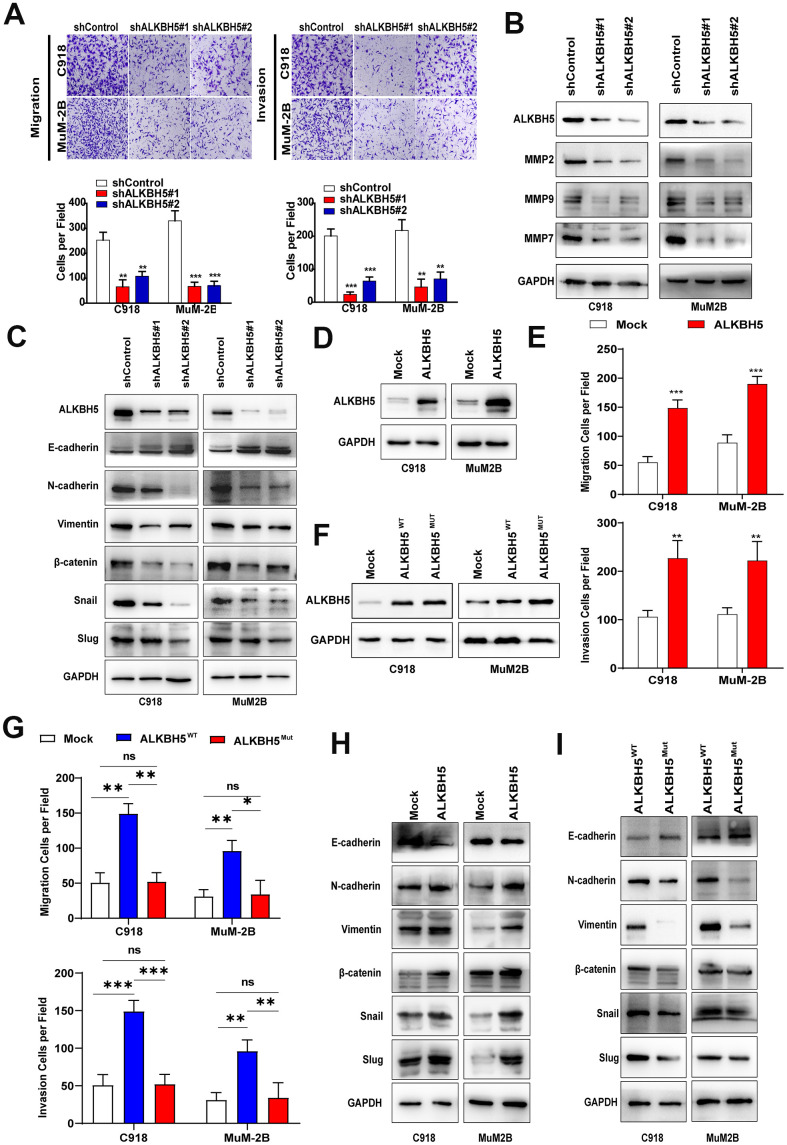
**ALKBH5 promoted UM cell migration, invasion, and EMT.** (**A**) After ALKBH5 knockdown plasmid transfection for 24 hours, the transwell migration assay and Matrigel invasion assay were used to determine cell migration and invasion ability, respectively, in C918 and MuM-2B cells. (**B**) After transfection with shRNA-targeted ALKBH5, the expression of MMP2, MMP7, and MMP9 was detected using western blot. (**C**) Knockdown of ALKBH5 decreased mesenchymal markers (N-cadherin, vimentin, Snail, Slug, and β-catenin) and increased epithelial marker (E-cadherin) in UM cells. (**D**) The ALKBH5 overexpression efficiency was verified at the protein level in UM cells by western blot assay. (**E**) Upregulation of ALKBH5 increased cell migration and invasion abilities in UM cells. (**F**) Western blot assay was used to detect the ALKBH5 protein level by transfecting UM cells with wild-type or catalytic inactive mutation plasmid of ALKBH5. (**G**) Compared with ALKBH5 wild-type plasmid transfection, catalytic inactive mutation of ALKBH5 decreased the migration and invasion of UM cells. (**H**) Overexpression of ALKBH5 increased the expression of EMT markers. (**I**) Loss of ALKBH5 catalytic activity suppressed the expression of EMT-related proteins in UM cells. Mean ± SEM, t-test, *P < 0.05, **P < 0.01, ***P < 0.001.

Transwell migration and Matrigel invasion assays were performed to evaluate the effects of ALKBH5 on cell migration and invasion. As shown in Figure 4A, knockdown of ALKBH5 significantly inhibited migration and invasion of MuM-2B and C918 cells. Western blot assay demonstrated that the downregulation of ALKBH5 reduced the expression of the tumor metastasis-related proteins matrix metallopeptidase 2 (MMP2), MMP7, and MMP9 in UM cells (Figure 4B). These findings indicate that ALKBH5 silencing reduces migration and invasion of UM cells.

### ALKBH5 promotes EMT of UM cells via m^6^A demethylation

Because EMT has been found to be a key process in tumor metastasis, we investigated the relationship between ALKBH5 and EMT in UM. As shown in Figure 4C, downregulation of ALKBH5 increased E-cadherin and decreased the protein levels of N-cadherin, Vimentin, Snail, Slug, and β-catenin (Figure 4C), which indicates that ALKBH5 affects the EMT of UM cells. In addition, overexpression of ALKBH5 not only significantly increased the migration and invasion of UM cells (Figure 4D, 4E, Supplementary Figure 2A), but also promoted UM cell EMT (Figure 4H). Moreover, IHC staining was used to detect EMT markers expression in tumors of nude mice assay of C918-shALKBH5#2 group and C918-shControl group. We found that Vimentin and Snail expressions were dramatically suppressed in the C918-shALKBH5#2 group than that in C918-shControl group (Supplementary Figure 2B).

ALKBH5, an m^6^A demethylase, can erase m^6^A mRNA modification and contribute to the progression of carcinomas [12]. Thus, we explored whether ALKBH5 upregulation and functional changes are a consequence of m^6^A demethylation. The reported catalytic inactive mutation ALKBH5 H204A (ALKBH5^MUT^) plasmid and the wild-type ALKBH5 (ALKBH5^WT^) plasmid were transfected into C918 and MuM-2B cells (Figure 4F). Compared with ALKBH5^MUT^, ectopic expression of ALKBH5^WT^ increased cell migration and invasion (Figure 4G and Supplementary Figure 2C). Furthermore, cells with the catalytic inactive mutation of ALKBH5 showed lower N-cadherin, Vimentin, Snail, Slug, and β-catenin expression and higher E-cadherin expression (Figure 4I). These results indicate that ALKBH5-induced m^6^A demethylation is critical to EMT in UM.

### ALKBH5-induced m^6^A demethylation increases FOXM1 expression in UM cells

ALKBH5 has been reported to demethylate FOXM1 nascent transcripts and promote FOXM1 expression in glioblastoma [23]. Moreover, ALKBH5 downregulates the m^6^A enrichment on FOXM1 mRNA and promotes cell proliferation and invasion in lung adenocarcinoma [24]. Therefore, we firstly used the GSE22138, GSE73652, and TCGA data sets to investigate the relationship between FOXM1 and ALKBH5 in UM. We found that ALKBH5 expression is positively correlated with FOXM1 expression in UM tumors (Supplementary Figure 3A–3C). In addition, IHC staining also demonstrated that knockdown ALKBH5 could decrease FOXM1 expression *in vivo* (Supplementary Figure 3D). Furthermore, higher FOXM1 expression predicted poor prognosis in patients with UM in the TCGA data set (Supplementary Figure 3E).

**Figure 5:**
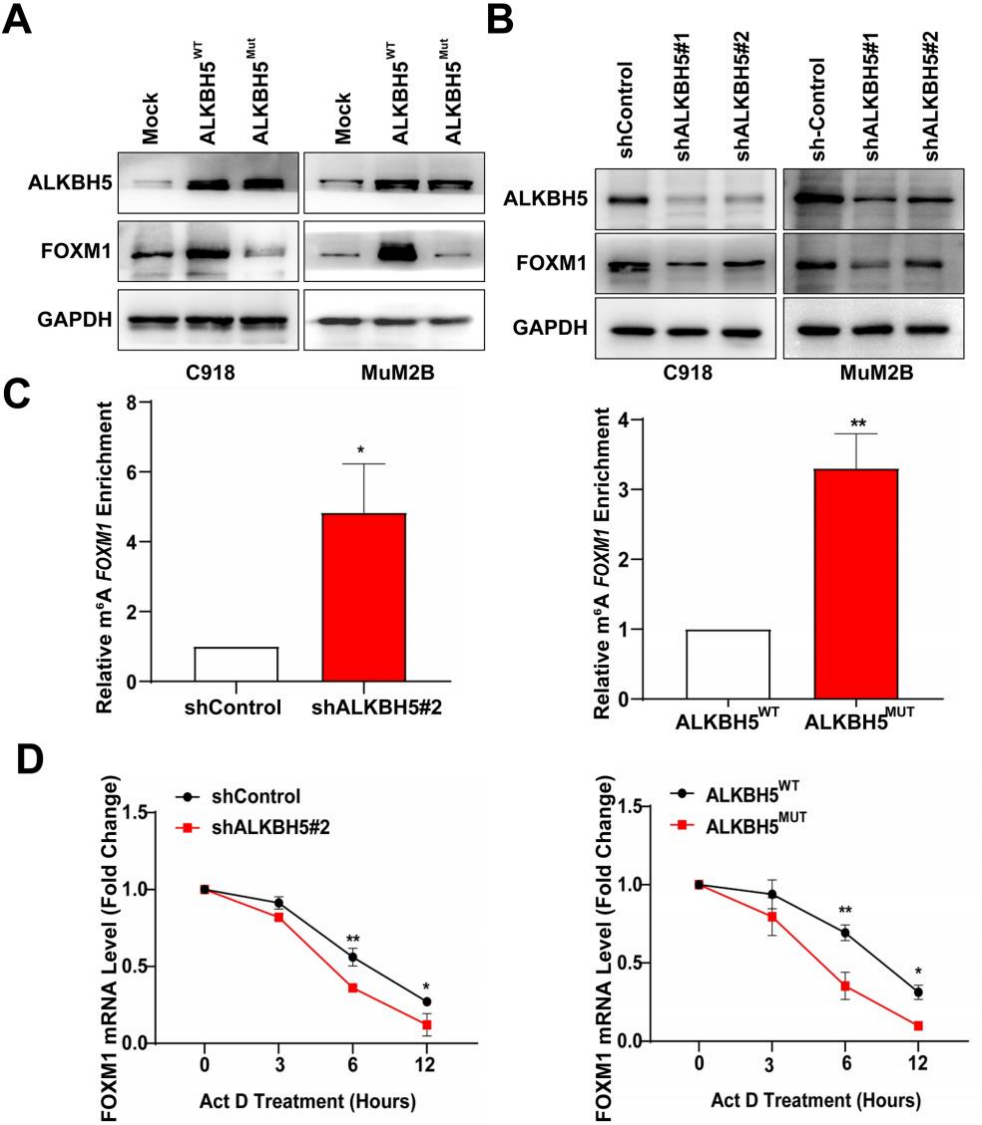
**ALKBH5 increases FOXM1 expression and *FOXM1* mRNA stability in UM cells.** (**A**) The protein levels of FOXM1 in wild-type or catalytic inactive mutation ALKBH5-expressing UM cells were measured using western blotting. (**B**) ALKBH5 downregulation decreased FOXM1 expression in UM cells. (**C**) MeRIP-qPCR analysis was used to verify ALKBH5-induced FOXM1 m^6^A modification. The m^6^A modification of FOXM1 was increased on downregulation and catalytic inactive mutation of ALKBH5 in C918 cells. (**D**) C918 cells were treated with dactinomycin (Act D, 2 μg/mL) to block new RNA synthesis. The stability of FOXM1 was measured by qRT-PCR at different times. Mean ± SEM, t-test, *P < 0.05, **P < 0.01, ***P < 0.001.

To further confirm whether ALKBH5 could regulate FOXM1 expression, western blot assay was performed to detect protein levels of FOXM1 after transfection of ALKBH5 treated with different shRNAs. We found that FOXM1 expression increased after transfecting UM cells with ALKBH5^WT^ plasmid but did not change after treating UM cells with the catalytic inactive mutation of ALKBH5 (Figure 5A). Moreover, knockdown of ALKBH5 decreased the expression of FOXM1 (Figure 5B). We also used the MeRIP-qPCR assay to detect the effect of ALKBH5 on m^6^A levels of FOXM1 mRNA. As shown in Figure 5C, the downregulation of ALKBH5 increased the m^6^A level of FOXM1 mRNA in C918 cells (Figure 5C, left panel). In addition, m^6^A levels of FOXM1 mRNA were significantly increased in cells transfected with ALKBH5^MUT^ plasmids compared with wild-type plasmids (Figure 5C, right panel). Our results indicated that ALKBH5 demethylates the m^6^A modification on FOXM1 mRNA in UM cells.

Investigators have found that ALKBH5-induced m^6^A demethylation on mRNAs affects mRNA stability and translation [25, 26]. To investigate whether ALKBH5 increases FOMX1 expression by increasing its mRNA stability, we treated cells with the transcription inhibitor dactinomycin and detected the level of FOXM1 mRNA. As shown in Figure 5D (left panel), knockdown of ALKBH5 in C918 cells significantly decreased the FOXM1 mRNA level compared with control cells. Furthermore, the level of FOXM1 mRNA was notably reduced in C918 cells with catalytic inactive mutation of ALKBH5 after dactinomycin treatment (Figure 5D, right panel). Thus, these results demonstrated that ALKBH5 may upregulate FOXM1 expression and increase the stability of FOXM1 mRNA via demethylating m^6^A modification.

### FOXM1 is involved in ALKBH5-induced EMT in UM cells

**Figure 6:**
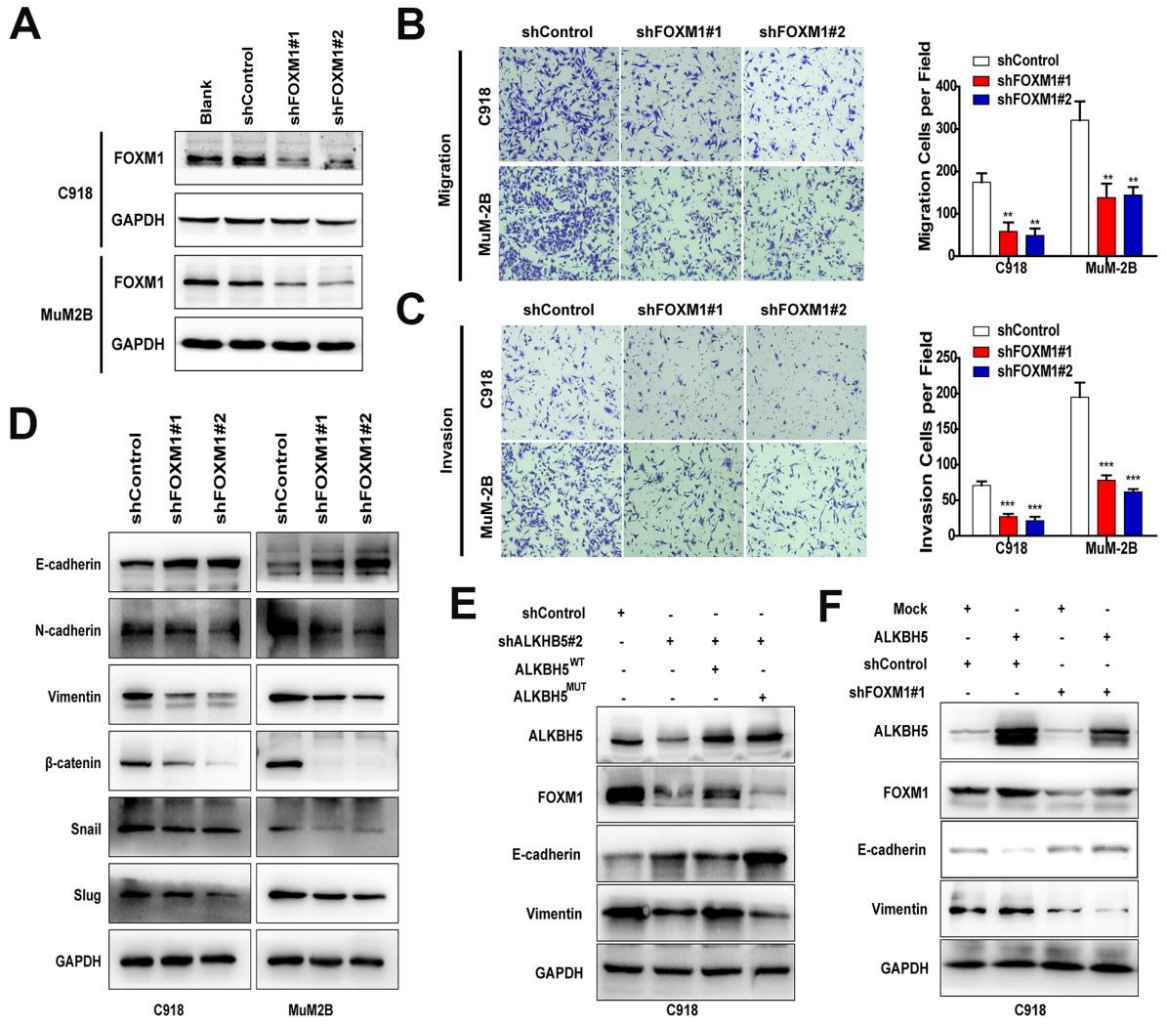
**FOXM1 is involved in ALKBH5-induced EMT in UM cells.** (**A**) The FOXM1 knockdown efficiency was verified at the protein level in UM cells by western blot assay. Downregulation of FOXM1 decreased cell migration (**B**) and invasion (**C**) of UM cells. (**D**) Downregulation of FOXM1 increased E-cadherin expression and decreased the protein levels of N-cadherin, vimentin, Snail, Slug, and β-catenin. (**E**) Western blot of FOXM1, E-cadherin, and vimentin in C918 cells with or without ALKBH5 stable knockdown transfected with wild-type or H204A mutation plasmid of ALKBH5. (**F**) The protein levels of E-cadherin and vimentin were measured using western blot in C918 cells stable transfected with shControl and shFOXM1 plasmid with or without ALKBH5 overexpression. Mean ± SEM, t-test, *P < 0.05, **P < 0.01, ***P < 0.001.

FOXM1 is critical to the EMT/mesenchymal-epithelial transition (MET) process in carcinomas [27], but its role in UM remains unknown. In this study, we designed two different shRNAs to target FOXM1 and confirmed the knockdown efficiency by western blot (Figure 6A). Downregulation of FOXM1 significantly suppressed cell migration (Figure 6B) and invasion (Figure 6C). In addition, inhibition of FOXM1 in UM cells increased E-cadherin expression and decreased N-cadherin, vimentin, Snail, Slug, and β-catenin expression (Figure 6D).

To verify that m^6^A modification of FOXM1 mRNA is critical for ALKBH5-induced EMT, ALKBH5^WT^ and ALKBH5^MUT^ plasmids were transfected into ALKBH5-stable knockdown C918 cells. As expected, FOXM1 expression and EMT were markedly rescued by overexpression of the wild-type ALKBH5 but not the catalytic inactive mutation (Figure 6E). In addition, FOXM1-stable knockdown C918 cells were transduced with ALKBH5 overexpression and Control plasmid. As shown in Figure 6F, overexpression of ALKBH5 cannot rescue the deceleration of EMT caused by FOXM1 downregulation. Furthermore, the migration and invasion abilities of FOXM1-knockdown cells could not be promoted by ALKBH5 overexpression (Supplementary Figure 4). Taken together, our results suggest that ALKBH5 may promote UM EMT by upregulating FOXM1 expression in an m^6^A modification–dependent manner.

## DISCUSSION

ALKBH5 has been shown to be involved in carcinogenesis and progression of a variety of cancers. However, the function of ALKBH5 in cancer is still controversial. In lung cancer [24, 28], gastric cancer [29], and epithelial ovarian cancer [30], ALKBH5 may act as an oncogene, whereas in colon cancer [31] and pancreatic cancer [32], ALKBH5 may inhibit tumor progression. However, few studies have investigated the role of ALKBH5 in UM. In this study, we demonstrated that inhibition of ALKBH5 suppresses tumor growth *in vivo* and that EP300-induced H3K27ac activation promotes ALKBH5 expression. A series of *in vitro* functional experiments verified that the downregulation of ALKBH5 inhibits cell proliferation, migration, and invasion and promotes cell apoptosis and G1 to S phase arrest. We also investigated the relationship between ALKBH5 and EMT. Western blot assay results showed that knockdown or loss of m^6^A catalytic activity of ALKBH5 increased the epithelial cell phenotype marker E-cadherin and decreased mesenchymal phenotype markers (N-cadherin and Vimentin) compared with controls. Furthermore, ALKBH5 increases the expression and mRNA stability of FOXM1, a transcription factor found to promote EMT, via m^6^A modification.

ALKBH5 has been reported to have many biological functions. In epithelial ovarian cancer, ALKBH5 inhibits cell autophagy by enhancing the expression and stability of BCL-2 through m^6^A modification of BCL-2 mRNA [30]. ALKBH5 inhibits tumor motility and stemness by demethylating TIMP3 [28], LncRNA NEAT1 [29], WIF1 [32], and LncRNA KCNK15-AS1 [33]. ALKBH5 regulates FOXM1 expression by demethylating the nascent transcripts of FOXM1 in glioblastoma stem-like cells [23]. However, in lung adenocarcinoma, ALKBH5 directly downregulates the m^6^A modification of FOXM1 mRNA and promotes FOXM1 expression under intermittent hypoxia [24]. In the present study, we demonstrated that ALKBH5 knockdown or loss of m^6^A catalytic activity had significantly decreased m^6^A enrichment of FOXM1 mRNA. Further studies should be performed to elucidate the oncogenic role of ALKBH5 in UM.

Although the function of ALKBH5 has been widely investigated, few studies focus on the regulatory mechanism of abnormal ALKBH5 expression. In breast cancer, ALKBH5 is a direct target of hypoxia-inducible factor 1α (HIF-1α) and HIF-2α and regulates breast cancer stem cell phenotype by downregulating the m^6^A modification on Nanog mRNA methylation [34]. It is well known that histone modifications involve a series of posttranslational modifications, including acetylation, methylation, and phosphorylation, that can impact gene expression by changing chromatin structure or recruiting histone modifiers [35, 36]. To further explore the relationship between histone modification and elevated ALKBH5 expression in UM, we analyzed the data from UCSC Genome Bioinformatics Site and found high enrichments of H3K27ac peaks at the promoter of ALKBH5. Although the levels of both EP300 and CREBBP (the members of the EP300/CBP complex) were positively correlated with ALKBH5 in UM, we verified that EP300-induced H3K27ac activation promotes ALKBH5 transcription and increases ALKBH5 expression in UM. Our results provide new insight into the mechanisms involved in ALKBH5-related tumor progression.

FOXM1 has been reported to be involved in various tumor processes, including cancer growth and aggression, cancer differentiation, stem cell phenotype, and EMT [37]. As a transcription factor, FOXM1 regulates the transcription of its downstream genes by directly binding to promoters of their DNA [27]. FOXM1 promotes EMT in pancreatic cancer by directly binding to the promoter region of the caveolin-1 gene and promoting its expression [38]. Snail and Slug, the transcription factors of EMT, are directly regulated by FOXM1 [39, 40]. In addition, FOXM1 not only regulates β-catenin expression but also reduces the nuclear accumulation and activity of β-catenin [41, 42]. In the current study, we observed that FOXM1 knockdown not only decreased the invasion and migration of UM cells, but also induced the downregulation of Snail, Slug, and β-catenin. In addition, the downregulation of FOXM1 in UM cells decreased the expression of the mesenchymal markers Vimentin and N-cadherin and increased the expression of the epithelial marker E-cadherin. Finally, the expression of FOXM1 was upregulated by ALKBH5-induced m^6^A demethylation, indicating that the ALKBH5/FOXM1 axis promotes UM EMT.

**Figure 7:**
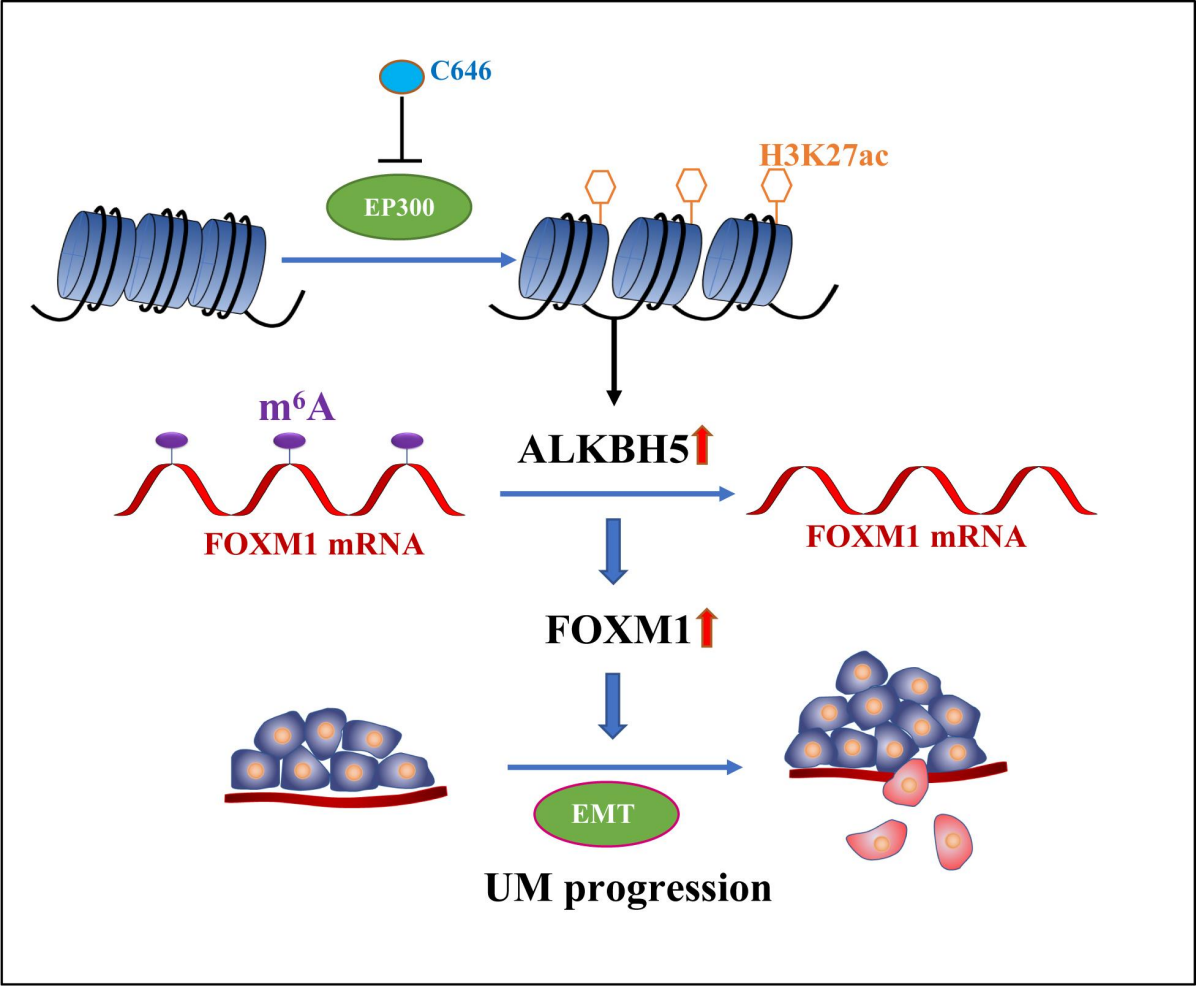
**The graphic illustration of ALKBH5-mediated m^6^A demethylation of *FOXM1* mRNA promotes progression of uveal melanoma.**

In conclusion, we demonstrate that ALKBH5, which is positively regulated by epigenetic modifications of H3K27 acetylation, promotes tumor progression by inducing tumor EMT and increasing FOXM1 expression via m^6^A demethylation (Figure 7). Therefore, ALKBH5 is a potential target of UM molecular therapy.

## MATERIALS AND METHODS

### Cell culture and reagent

A human retinal pigment epithelium cell line (ARPE-19) was obtained from ATCC (ATCC CRL2302, Manassas, VA). Human UM cell lines (MuM-2B and C918) were obtained from the Cell Resource Center, Peking Union Medical College (Beijing, China). All cell lines were cultured in RPMI-1640 medium with supplementation of 10% fetal bovine serum (FBS; Invitrogen, Carlsbad, CA) and appropriate amounts of penicillin (100 U/mL) and streptomycin (100 mg/mL) in a humidified atmosphere of 5% CO_2_ at 37° C. The histone acetyltransferase inhibitors C646, which targets EP300, and SGC-CBP30, which targets cAMP response element binding protein (CREB) binding protein (CREBBP), were purchased from Selleck (Shanghai, China).

### Western blot

Proteins were collected from cells using the Whole Cell Lysis Assay Kit (KGP250, KeyGen, Nanjing, China). The protein concentration was determined using the bicinchoninic acid (BCA) method using the BCA Protein Quantitation Assay Kit (KeyGen, Nanjing, China). Protein was electrophoretically separated by 10% or 15% SDS-PAGE and transferred to PVDF membranes (Millipore, Billerica, MA). The membranes were blocked for 1 hour with 5% bovine serum albumin (BSA) in TBS-T and incubated with specific primary antibodies overnight at 4° C followed by incubation with rabbit or mouse radish peroxidase-coupled secondary antibodies for 1 hour. Antibody binding was detected using the enhanced chemiluminescence reagent (Millipore, Billerica, MA). The antibodies used in this study were as follows: ALKBH5 (ab195377, Abcam), H3K27ac (#8173, Cell Signaling Technology [CST]), cyclin D1 (#2978, CST), cyclin E1 (ab33911, Abcam), c-Myc (#5605, CST), caspase-3 (19677-1-AP, Proteintech), BCL-2 (12789-1-AP, Proteintech), MMP2 (#87809, CST), MMP7 (#71031, CST), MMP9 (#13667, CST), E-cadherin (#3195, CST), N-cadherin (#13116, CST), vimentin (#5741, CST), β-catenin (#8480, CST), Snail (ab53519, Abcam), Slug (ab27568, Abcam), β-actin (ab8227, Abcam), and GAPDH (#8884, CST).

### Immunohistochemical staining

For immunohistochemical staining, deparaffinized sections were pretreated with 10 mM of sodium citrate buffer for antigen unmasking, blocked with 5% goat serum for 30 minutes, and incubated with antibodies at 4° C overnight. IHC staining was performed with horseradish peroxidase (HRP) conjugates using DAB, and the nuclei were stained with hematoxylin. Images were taken with a Nikon microscopy and K-viewer system.

### Plasmid construction and transfection

The shRNA sequences of ALKBH5 are as follows: shALKBH5#1, 5′-GAAAGGCTGTTGGCATCAATA-3′; shALKBH5#2, 5′-CCTCAGGAAGACAAGATTAGA-3′. The downregulation target sequences of FOXM1 are as follows: shFOXM1#1, 5′-CTCTTCTCCCTCAGATATA-3′; shFOXM1#2, 5′-GGACCACTTTCCCTACTTT-3′. The catalytic inactive mutation site of ALKBH5 H204A was obtained from a previously published study [23]. Plasmids were transfected with Lipofectamine 2000 (Invitrogen, Carlsbad, CA) according to the manufacturer’s instructions.

### Lentiviral transduction for stable cell lines

Lentivirus plasmids of shRNA were co-transfected with the packaging plasmids pMD2.G and psPAX2 (Addgene) into HEK-293T cells to produce lentivirus. Two days after transfection, virus supernatants were collected, concentrated, and used to infect UM cells with polybrene (8 μg/mL, Sigma, St. Louis, MO). After 3 days of transduction, cells were treated with 2 μg/mL of puromycin (Sigma) for 4 to 7 days.

### Quantitative real-time polymerase chain reaction (qRT-PCR)

Total RNA was isolated using RNA plus reagent (Takara, Japan). cDNA was prepared using Oligo(dT) primers according to the protocol supplied with the PrimeScript RT Reagent (Takara, Japan). The expression of ALKBH5 was determined by qRT-PCR using TB Green Advantage qPCR Premix (Takara, Japan). The primer sequences of ALKBH5, FOXM1, and GAPDH used in this study were as follows: ALKBH5, forward: 5′-GGTGTCGGAACCAGTGCTTT-3′, reverse: 3′-CCAACCGGGGTGCATCTAAT-5′; FOXM1, forward: 5′-ACGTCCCCAAGCCAGGCTC-3′, reverse: 5′-CTATGTAGCTCAGGAATAA-3′; GAPDH, forward: 5′-ACAACTTTGGTATCGTGGAAGG-3′, reverse: 3′-GCCATCACGCCACAGTTTC-5′.

### Proliferation assay

Cell proliferation was assessed using the 3-(4,5-dimethylthiazol-2-yl)-2, 5-diphenyltetrazolium bromide (MTT) assay. Cells were plated into 96-cell plates (1000 cells per well). MTT solution (5 mg/mL) was added to the medium of each well. After incubation for 4 hours, the resultant formazan crystals were dissolved in dimethyl sulfoxide, and the absorbance intensity was measured using a microplate reader at 492 nm.

### Migration and invasion assay

Cell migration assays were performed using 24-well transwell chambers (Costar-Corning, New York, USA) with an 8.0-μm pore polycarbonate filter. The lower chamber was filled with 700 μL of RPMI-1640 with 10% FBS, and cells (2.5 × 104 cells/well) pretreated with plasmid transfection of ALKBH5 were added into the upper chamber. After incubation for 5 hours, cells were fixed with methanol, stained with crystal violet, photographed, and counted. Cell invasion assay was performed similarly, except that transwell inserts were precoated with Matrigel and incubated for 10 hours.

### Apoptosis and cell cycle assay

For cell apoptosis analysis, cells were serum-starved overnight and determined using the Annexin V-FITC Apoptosis Detection Kit (Beyotime, Shanghai, China). For cell cycle analysis, cells were fixed in 70% ethanol, stained with propidium iodide, and analyzed by flow cytometry.

### m^6^A-RNA immunoprecipitation (MeRIP) assay

Total RNAs were extracted from stable ALKBH5 knockdown and catalytic inactive mutation C918 cells and their corresponding control cells. Chemically fragmented RNA (approximately 100 nucleotides) was incubated with m^6^A antibody (New England BioLabs) for immunoprecipitation according to the protocol of the Magna methylated RNA immunoprecipitation m^6^A kit (Merck Millipore). Enrichment of m^6^A-containing mRNA was analyzed using qRT-PCR.

### Dactinomycin treatment

C918 cells were exposed to 2 μg/mL of dactinomycin (Selleck, Shanghai, China) to block transcription for 0, 3, 6, and 12 hours. The cells were harvested, and the stability of *FOXM1* mRNA was analyzed using qRT-PCR assay.

### Chromatin immunoprecipitation (ChIP)

ChIP analysis was carried out according to the standard method of the Simple ChIP Enzymatic Chromatin IP Kit (#9003, Cell Signaling Technology). Chromatin was immunoprecipitated with anti-H3K27ac (#8173, Cell Signaling Technology) or anti-IgG as a negative control. Finally, immunoprecipitated protein and DNA complexes were isolated and RT-PCR assay was carried out to examine the quantity of the specific proteins. The primers for the ALKBH5 promoters are listed as following: ALKBH5, forward: 5′-CGCGGGTTTCAGAACTTTCC-3′, reverse: 3′-GGAGTTTCCGGAAGTCGGTT-5′.

### Mice xenograft model

Animal experiments were approved by the Ethics Committee of Jinan University. The *in vivo* experiment method for transplantation of tumors was subcutaneous injection of 1 × 10^7^ ALKBH5-stable knockdown C918 cells into BALB/c nude mice. The length and width of tumors were measured every 3 days to determine the tumor volume. After 4 weeks, the tumor-bearing mice were sacrificed, and the tumors were weighed.

### Bioinformatics analysis

The gene expression profile GSE22138 and GSE73652 data sets were downloaded from the database and analyzed using R (V4.0, https://cran.r-project.org/) with the GEOquery Package. The GEPIA database (http://gepia.cancer-pku.cn/index.html) was used to analyze the interaction between ALKBH5, FOXM1, and clinical characteristics.

### Statistical analysis

Data were analyzed for statistical significance using the t-test (IBM, NY). In these analyses, *P* < 0.05 was considered statistically significant. All *in vitro* experiments were confirmed three times.

## Supplementary Material

Supplementary Figures

## References

[1] Kaliki S, Shields CL. Uveal melanoma: relatively rare but deadly cancer. Eye (Lond). 2017; 31:241–57. 10.1038/eye.2016.275 27911450 PMC5306463

[2] Krantz BA, Dave N, Komatsubara KM, Marr BP, Carvajal RD. Uveal melanoma: epidemiology, etiology, and treatment of primary disease. Clin Ophthalmol. 2017; 11:279–89. 10.2147/OPTH.S89591 28203054 PMC5298817

[3] Carvajal RD, Schwartz GK, Tezel T, Marr B, Francis JH, Nathan PD. Metastatic disease from uveal melanoma: treatment options and future prospects. Br J Ophthalmol. 2017; 101:38–44. 10.1136/bjophthalmol-2016-309034 27574175 PMC5256122

[4] Rietschel P, Panageas KS, Hanlon C, Patel A, Abramson DH, Chapman PB. Variates of survival in metastatic uveal melanoma. J Clin Oncol. 2005; 23:8076–80. 10.1200/JCO.2005.02.6534 16258106

[5] Lorenzo D, Ochoa M, Piulats JM, Gutiérrez C, Arias L, Català J, Grau M, Peñafiel J, Cobos E, Garcia-Bru P, Rubio MJ, Padrón-Pérez N, Dias B, et al. Prognostic factors and decision tree for long-term survival in metastatic uveal melanoma. Cancer Res Treat. 2018; 50:1130–39. 10.4143/crt.2017.171 29198096 PMC6192913

[6] Helgadottir H, Höiom V. The genetics of uveal melanoma: current insights. Appl Clin Genet. 2016; 9:147–55. 10.2147/TACG.S69210 27660484 PMC5019476

[7] Robertson AG, Shih J, Yau C, Gibb EA, Oba J, Mungall KL, Hess JM, Uzunangelov V, Walter V, Danilova L, Lichtenberg TM, Kucherlapati M, Kimes PK, et al, and TCGA Research Network. Integrative Analysis Identifies Four Molecular and Clinical Subsets in Uveal Melanoma. Cancer Cell. 2017; 32:204–220.e15. 10.1016/j.ccell.2017.07.003 28810145 PMC5619925

[8] Sharma A, Stei MM, Fröhlich H, Holz FG, Loeffler KU, Herwig-Carl MC. Genetic and epigenetic insights into uveal melanoma. Clin Genet. 2018; 93:952–61. 10.1111/cge.13136 28902406

[9] Herlihy N, Dogrusöz M, van Essen TH, Harbour JW, van der Velden PA, van Eggermond MC, Haasnoot GW, van den Elsen PJ, Jager MJ. Skewed expression of the genes encoding epigenetic modifiers in high-risk uveal melanoma. Invest Ophthalmol Vis Sci. 2015; 56:1447–58. 10.1167/iovs.14-15250 25593028 PMC5102441

[10] Li Y, Sun W, Sun D, Yin D. Ras-ERK1/2 signaling promotes the development of uveal melanoma by downregulating H3K14ac. J Cell Physiol. 2019; 234:16011–20. 10.1002/jcp.28259 30770563

[11] Lee Y, Choe J, Park OH, Kim YK. Molecular mechanisms driving mRNA degradation by m^6^A modification. Trends Genet. 2020; 36:177–88. 10.1016/j.tig.2019.12.007 31964509

[12] Huang H, Weng H, Chen J. The biogenesis and precise control of RNA m^6^A methylation. Trends Genet. 2020; 36:44–52. 10.1016/j.tig.2019.10.011 31810533 PMC6925345

[13] Ma S, Chen C, Ji X, Liu J, Zhou Q, Wang G, Yuan W, Kan Q, Sun Z. The interplay between m^6^A RNA methylation and noncoding RNA in cancer. J Hematol Oncol. 2019; 12:121. 10.1186/s13045-019-0805-7 31757221 PMC6874823

[14] Jia R, Chai P, Wang S, Sun B, Xu Y, Yang Y, Ge S, Jia R, Yang YG, Fan X. m^6^A modification suppresses ocular melanoma through modulating HINT2 mRNA translation. Mol Cancer. 2019; 18:161. 10.1186/s12943-019-1088-x 31722709 PMC6854757

[15] Luo G, Xu W, Zhao Y, Jin S, Wang S, Liu Q, Chen X, Wang J, Dong F, Hu DN, Reinach PS, Yan D. RNA m^6^A methylation regulates uveal melanoma cell proliferation, migration, and invasion by targeting c-Met. J Cell Physiol. 2020; 235:7107–19. 10.1002/jcp.29608 32017066

[16] Schulz M, Salamero-Boix A, Niesel K, Alekseeva T, Sevenich L. Microenvironmental regulation of tumor progression and therapeutic response in brain metastasis. Front Immunol. 2019; 10:1713. 10.3389/fimmu.2019.01713 31396225 PMC6667643

[17] Aiello NM, Kang Y. Context-dependent EMT programs in cancer metastasis. J Exp Med. 2019; 216:1016–26. 10.1084/jem.20181827 30975895 PMC6504222

[18] Lin X, Chai G, Wu Y, Li J, Chen F, Liu J, Luo G, Tauler J, Du J, Lin S, He C, Wang H. RNA m^6^A methylation regulates the epithelial mesenchymal transition of cancer cells and translation of snail. Nat Commun. 2019; 10:2065. 10.1038/s41467-019-09865-9 31061416 PMC6502834

[19] Yue B, Song C, Yang L, Cui R, Cheng X, Zhang Z, Zhao G. METTL3-mediated N6-methyladenosine modification is critical for epithelial-mesenchymal transition and metastasis of gastric cancer. Mol Cancer. 2019; 18:142. 10.1186/s12943-019-1065-4 31607270 PMC6790244

[20] Gong C, Shen J, Fang Z, Qiao L, Feng R, Lin X, Li S. Abnormally expressed JunB transactivated by IL-6/STAT3 signaling promotes uveal melanoma aggressiveness via epithelial-mesenchymal transition. Biosci Rep. 2018; 38:BSR20180532. 10.1042/BSR20180532 29899166 PMC6028753

[21] Duan F, Lin M, Li C, Ding X, Qian G, Zhang H, Ge S, Fan X, Li J. Effects of inhibition of hedgehog signaling on cell growth and migration of uveal melanoma cells. Cancer Biol Ther. 2014; 15:544–59. 10.4161/cbt.28157 24553082 PMC4026077

[22] Raisner R, Kharbanda S, Jin L, Jeng E, Chan E, Merchant M, Haverty PM, Bainer R, Cheung T, Arnott D, Flynn EM, Romero FA, Magnuson S, Gascoigne KE. Enhancer activity requires CBP/P300 bromodomain-dependent histone H3K27 acetylation. Cell Rep. 2018; 24:1722–29. 10.1016/j.celrep.2018.07.041 30110629

[23] Zhang S, Zhao BS, Zhou A, Lin K, Zheng S, Lu Z, Chen Y, Sulman EP, Xie K, Bögler O, Majumder S, He C, Huang S. m^6^A demethylase ALKBH5 maintains tumorigenicity of glioblastoma stem-like cells by sustaining FOXM1 expression and cell proliferation program. Cancer Cell. 2017; 31:591–606.e6. 10.1016/j.ccell.2017.02.013 28344040 PMC5427719

[24] Chao Y, Shang J, Ji W. ALKBH5-m^6^A-FOXM1 signaling axis promotes proliferation and invasion of lung adenocarcinoma cells under intermittent hypoxia. Biochem Biophys Res Commun. 2020; 521:499–506. 10.1016/j.bbrc.2019.10.145 31677788

[25] Li XC, Jin F, Wang BY, Yin XJ, Hong W, Tian FJ. The m^6^A demethylase ALKBH5 controls trophoblast invasion at the maternal-fetal interface by regulating the stability of CYR61 mRNA. Theranostics. 2019; 9:3853–65. 10.7150/thno.31868 31281518 PMC6587351

[26] Tang C, Klukovich R, Peng H, Wang Z, Yu T, Zhang Y, Zheng H, Klungland A, Yan W. ALKBH5-dependent m^6^A demethylation controls splicing and stability of long 3'-UTR mRNAs in male germ cells. Proc Natl Acad Sci USA. 2018; 115:E325–33. 10.1073/pnas.1717794115 29279410 PMC5777073

[27] Bella L, Zona S, Nestal de Moraes G, Lam EW. FOXM1: a key oncofoetal transcription factor in health and disease. Semin Cancer Biol. 2014; 29:32–39. 10.1016/j.semcancer.2014.07.008 25068996

[28] Zhu Z, Qian Q, Zhao X, Ma L, Chen P. N^6^-methyladenosine ALKBH5 promotes non-small cell lung cancer progress by regulating TIMP3 stability. Gene. 2020; 731:144348. 10.1016/j.gene.2020.144348 31927006

[29] Zhang J, Guo S, Piao HY, Wang Y, Wu Y, Meng XY, Yang D, Zheng ZC, Zhao Y. ALKBH5 promotes invasion and metastasis of gastric cancer by decreasing methylation of the lncRNA NEAT1. J Physiol Biochem. 2019; 75:379–89. 10.1007/s13105-019-00690-8 31290116 PMC6728298

[30] Zhu H, Gan X, Jiang X, Diao S, Wu H, Hu J. ALKBH5 inhibited autophagy of epithelial ovarian cancer through miR-7 and BCL-2. J Exp Clin Cancer Res. 2019; 38:163. 10.1186/s13046-019-1159-2 30987661 PMC6463658

[31] Yang P, Wang Q, Liu A, Zhu J, Feng J. ALKBH5 holds prognostic values and inhibits the metastasis of colon cancer. Pathol Oncol Res. 2020; 26:1615–23. 10.1007/s12253-019-00737-7 31506804

[32] Tang B, Yang Y, Kang M, Wang Y, Wang Y, Bi Y, He S, Shimamoto F. m^6^A demethylase ALKBH5 inhibits pancreatic cancer tumorigenesis by decreasing WIF-1 RNA methylation and mediating Wnt signaling. Mol Cancer. 2020; 19:3. 10.1186/s12943-019-1128-6 31906946 PMC6943907

[33] He Y, Hu H, Wang Y, Yuan H, Lu Z, Wu P, Liu D, Tian L, Yin J, Jiang K, Miao Y. ALKBH5 inhibits pancreatic cancer motility by decreasing long non-coding RNA KCNK15-AS1 methylation. Cell Physiol Biochem. 2018; 48:838–46. 10.1159/000491915 30032148

[34] Zhang C, Samanta D, Lu H, Bullen JW, Zhang H, Chen I, He X, Semenza GL. Hypoxia induces the breast cancer stem cell phenotype by HIF-dependent and ALKBH5-mediated m⁶A-demethylation of NANOG mRNA. Proc Natl Acad Sci USA. 2016; 113:E2047–56. 10.1073/pnas.1602883113 27001847 PMC4833258

[35] Dutta A, Abmayr SM, Workman JL. Diverse activities of histone acylations connect metabolism to chromatin function. Mol Cell. 2016; 63:547–52. 10.1016/j.molcel.2016.06.038 27540855 PMC5298895

[36] Zhao S, Zhang X, Li H. Beyond histone acetylation-writing and erasing histone acylations. Curr Opin Struct Biol. 2018; 53:169–77. 10.1016/j.sbi.2018.10.001 30391813

[37] Nandi D, Cheema PS, Jaiswal N, Nag A. FoxM1: repurposing an oncogene as a biomarker. Semin Cancer Biol. 2018; 52:74–84. 10.1016/j.semcancer.2017.08.009 28855104

[38] Huang C, Qiu Z, Wang L, Peng Z, Jia Z, Logsdon CD, Le X, Wei D, Huang S, Xie K. A novel FoxM1-caveolin signaling pathway promotes pancreatic cancer invasion and metastasis. Cancer Res. 2012; 72:655–65. 10.1158/0008-5472.CAN-11-3102 22194465 PMC3271134

[39] Yang C, Chen H, Tan G, Gao W, Cheng L, Jiang X, Yu L, Tan Y. FOXM1 promotes the epithelial to mesenchymal transition by stimulating the transcription of slug in human breast cancer. Cancer Lett. 2013; 340:104–12. 10.1016/j.canlet.2013.07.004 23856032

[40] Wei P, Zhang N, Wang Y, Li D, Wang L, Sun X, Shen C, Yang Y, Zhou X, Du X. FOXM1 promotes lung adenocarcinoma invasion and metastasis by upregulating SNAIL. Int J Biol Sci. 2015; 11:186–98. 10.7150/ijbs.10634 25561901 PMC4279094

[41] Jin B, Wang C, Li J, Du X, Ding K, Pan J. Anthelmintic niclosamide disrupts the interplay of p65 and FOXM1/β-catenin and eradicates leukemia stem cells in chronic myelogenous leukemia. Clin Cancer Res. 2017; 23:789–803. 10.1158/1078-0432.CCR-16-0226 27492973

[42] Chen Y, Li Y, Xue J, Gong A, Yu G, Zhou A, Lin K, Zhang S, Zhang N, Gottardi CJ, Huang S. Wnt-induced deubiquitination FoxM1 ensures nucleus β-catenin transactivation. EMBO J. 2016; 35:668–84. 10.15252/embj.201592810 26912724 PMC4801947

