# Supplementary material for: ALKBH5-mediated m6A demethylation of FOXM1 mRNA promotes progression of uveal melanoma
Source: Aging (Albany NY). 2021 Jan 10;13(3):4045–62. doi: 10.18632/aging.202371 (PMC7906204; doi:10.18632/aging.202371)
Supplement: Supplementary Figures [file aging-13-202371-s001.pdf]

SUPPLEMENTARY FIGURES

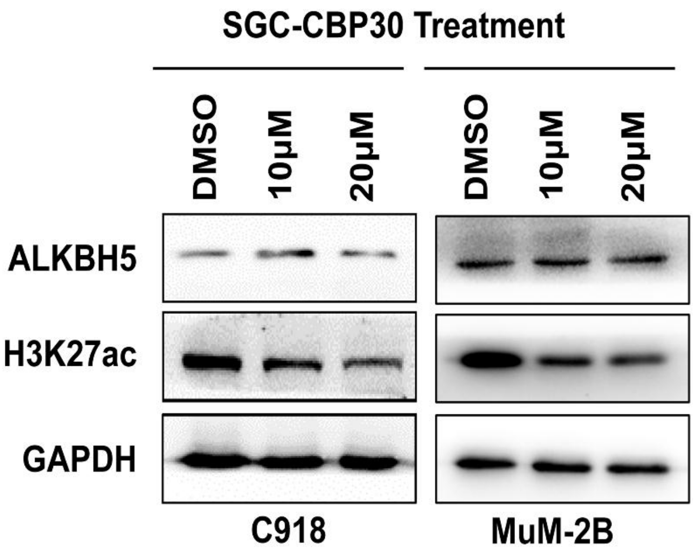

Supplementary Figure 1. After treating cells with SGC-CBP30 for 24 hours at different concentrations, the protein levels of ALKBH5 and H3K27ac were detected using western blot.

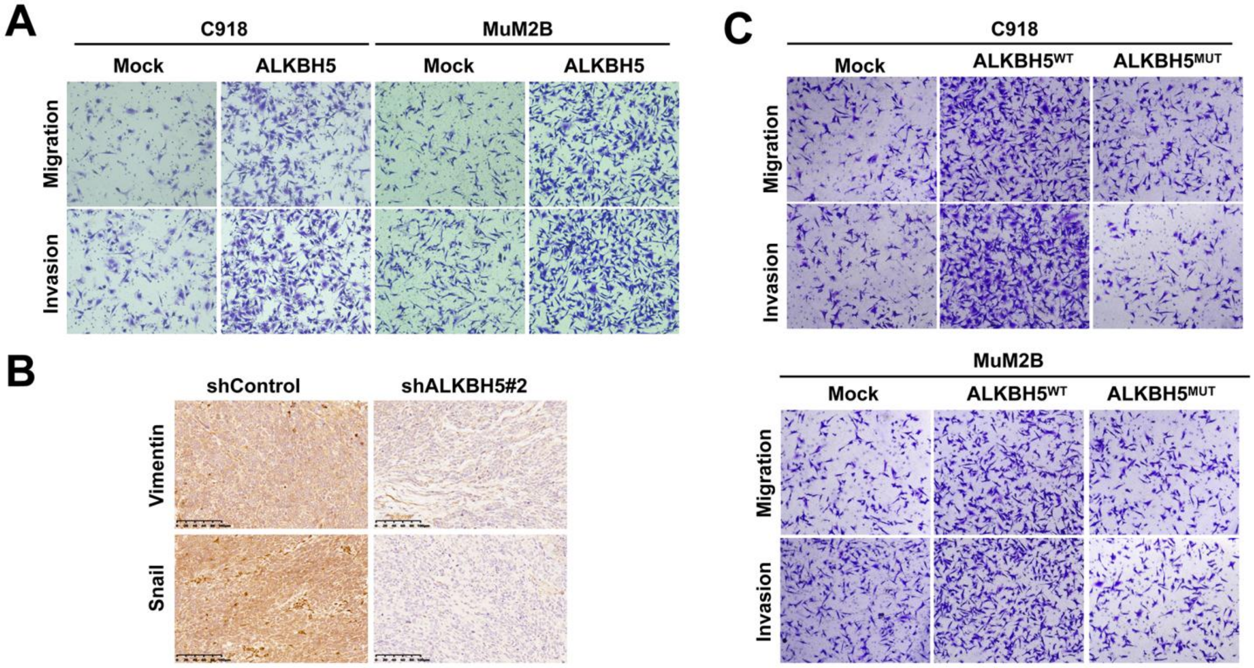

Supplementary Figure 2. ALKBH5 overexpression increased the abilities of migration and invasion in UM cells. (**A**) Upregulation of ALKBH5 increased cell migration and invasion in UM cells. (**B**) Vimentin and Snail expression were detected in C918-shALKBH5#2 and shcontrol group nude mice by IHC assay. (**C**) Compared with ALKBH5 wild-type plasmid transfection, cells with loss of catalytic activation ability of ALKBH5 could decrease the abilities of cell migration and invasion in UM.

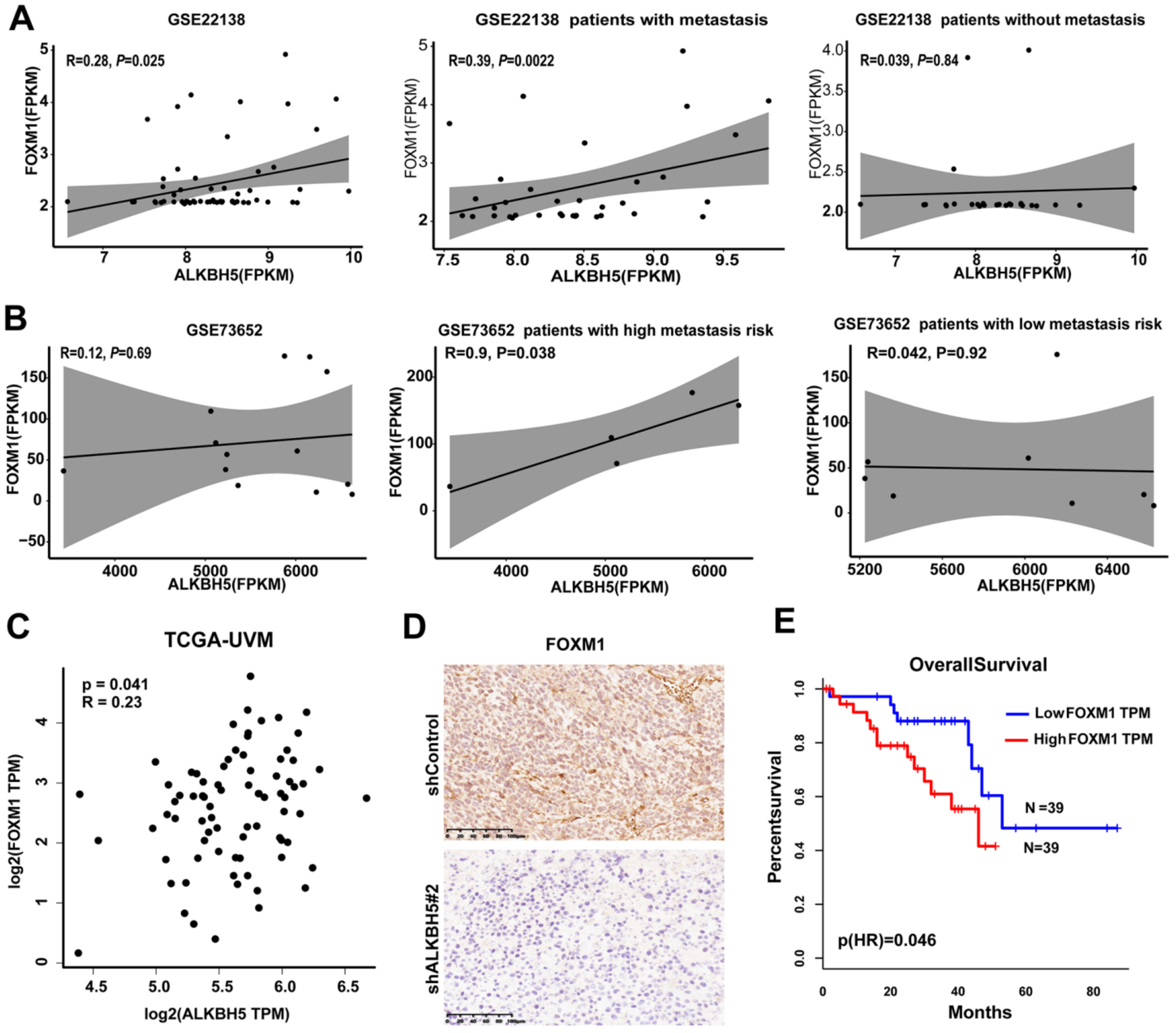

Supplementary Figure 3. Public data on UM tissues were used to analyze the relationship between ALKBH5 and FOXM1. (**A**) ALKBH5 expression was significantly correlated with FOXM1 expression (P = 0.025) in the GSE22138 data set (left panel). ALKBH5 expression in primary tumors with distant metastasis was positively correlated with FOXM1 expression (middle panel). ALKBH5 expression in primary tumors without distant metastasis was not significantly related to FOXM1 expression in the GSE22138 data set (right panel). (**B**) Although ALKBH5 was not correlated with FOXM1 in the GSE73652 data set (left panel), its expression showed a positive correlation in the primary tumors of patients with high metastasis risk (middle panel), but not in the primary tumors of patients with low metastasis risk (right panel). (**C**) In the TCGA database of UM tissue, ALKBH5 expression was positively related to FOXM1 expression. (**D**) The expression of FOXM1 was detected in the C918-shALKBH5#2 group and the control group by IHC assay. (**E**) Patients with higher FOXM1 expression have worse outcomes compared with those with low FOXM1 expression.

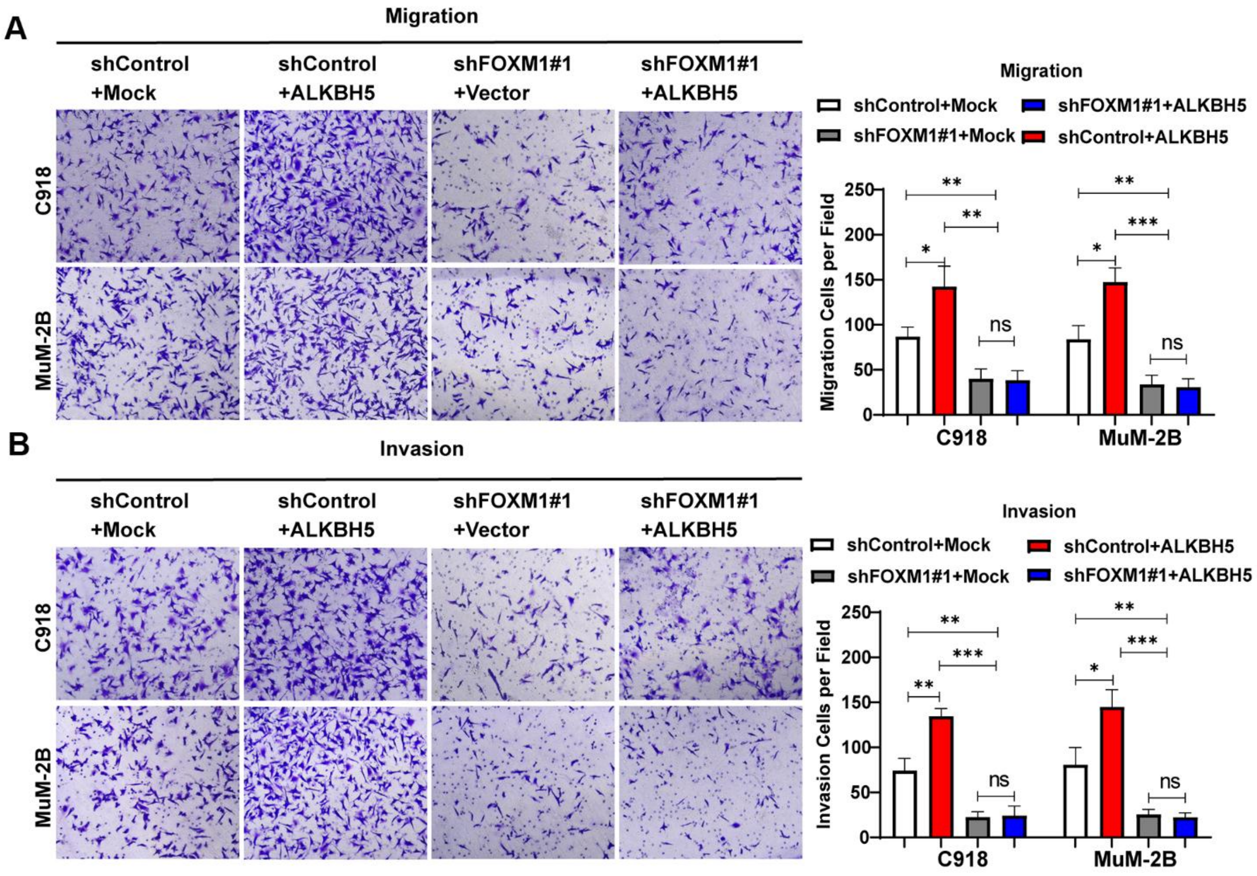

Supplementary Figure 4. ALKBH5 could not rescue cell Migration (**A**) and Invasion (**B**) abilities caused by FOXM1 knockdown in UM cells. Mean ± SEM, t-test, **P* < 0.05, ***P* < 0.01, ****P* < 0.001.
